# Identification and structural analysis of a thermophilic β-1,3-glucanase from compost

**DOI:** 10.1186/s40643-021-00449-4

**Published:** 2021-10-17

**Authors:** Jianwei Feng, Shenyuan Xu, Ruirui Feng, Andrey Kovalevsky, Xia Zhang, Dongyang Liu, Qun Wan

**Affiliations:** 1 College of Science, Nanjing Agricultural University, Nanjing, 210095 People’s Republic of China; 2 Key Laboratory of Plant Immunity, Nanjing Agricultural University, Nanjing, 210095 People’s Republic of China; 3 Key Laboratory of Bioorganic Synthesis of Zhejiang Province, College of Biotechnology and Bioengineering, Zhejiang University of Technology, Hangzhou, People’s Republic of China; 4 Neutron Scattering Division, Oak Ridge National Laboratory, Oak Ridge, TN 37831 USA; 5 Department of Molecular Biology, Qingdao Vland Biotech Group Inc., Qingdao, Shandong 266000 People’s Republic of China; 6 College of Resources and Environmental Sciences, Nanjing Agricultural University, Nanjing, 210095 People’s Republic of China

**Keywords:** β-1,3-glucanase, Crystal structure, Disulfide bond, Mutagenesis, Molecular dynamics

## Abstract

β-1,3-glucanase can specifically hydrolyze glucans to oligosaccharides and has potential applications in biotechnology. We used the metatranscriptomic technology to discover a thermophilic β-1,3-glucanase from compost. The phylogenetic study shows that it belongs to the family 16 glycoside hydrolase (GH16) and is most homologous with an enzyme from *Streptomyces sioyaensis*, an actinobacterium. It has the activity of 146.9 U/mg in the optimal reaction condition (75 °C and pH 5.5). Its catalytic domain was crystallized and diffracted to 1.14 Å resolution. The crystal structure shows a sandwich-like β-jelly-roll fold with two disulfide bonds. After analyzing the occurring frequencies of these cysteine residues, we designed two mutants (C160G and C180I) to study the role of these disulfide bonds. Both mutants have decreased their optimal temperature from 75 to 70 °C, which indicate that the disulfide bonds are important to maintain thermostability. Interestingly, the activity of C160G has increased ~ 17% to reach 171.4 U/mg. We speculate that the increased activity of C160G mutant is due to increased dynamics near the active site. Our studies give a good example of balancing the rigidity and flexibility for enzyme activity, which is helpful for protein engineering.

**Supplementary Information:**

The online version contains supplementary material available at 10.1186/s40643-021-00449-4.

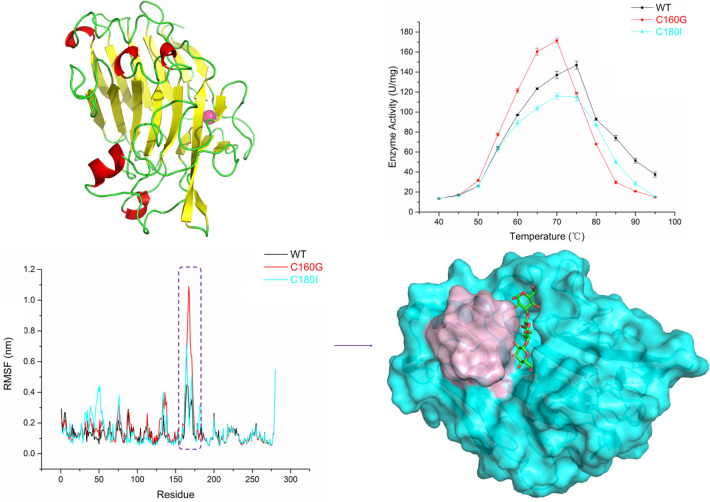

## Introduction

β-1,3-glucan is a non-starch polysaccharide consisting of glucose subunits connected through the β-1,3 glycosidic bonds. It is one of the major components of the cell wall of plant, fungi, and marine macroalgae (Zhu et al. 2015; Fibriansah et al. 2007). β-1,3-glucanase can specifically hydrolyze the glycosidic bonds to produce oligo- and monosaccharides, and has wide applications. For example, it can effectively inhibit the production of slime during the brewing process (Stahmann et al. 1993). It has potential usage to decompose algal biomass, which contains a large amount of β-1,3-glucan (Labourel et al. 2014). Discovering a highly active β-1,3-glucanase is essential.

Thermophilic enzymes have the optimal reaction temperatures above 60 °C (Singh et al. 2021; Niu et al. 2017). Compared with mesophilic enzymes, thermophilic enzymes are more stable and can be used for a longer time, which reduces the enzyme cost in biotechnology (Zhu et al. 2020). In addition, the high temperature environment can minimize contamination of polluted microorganisms in biotechnological processing (Laman and Youk 2020; Wang et al. 2020; Singh et al. 2021). However, many thermophilic enzymes have low catalytic activities, which makes them difficult to be applied in industries (Masuda et al. 2006; Kobayashi et al. 2016). Thus, discovering thermophilic enzymes with high activity is essential for biotechnology industries.

Composting is a biochemical process, during which environmental wastes, such as straw and animal manure, are mixed with thermophilic microorganisms to ferment and break down these organic wastes (Gurtler et al. 2018; Reyes-Torres et al. 2018). Composting has three phases: the mesophilic phase, the thermophilic phase, and the mature phase. In the thermophilic phase, there are lots of thermally stable enzymes secreted by either introduced or natural microorganisms (Wang et al. 2021). Though many natural microorganisms in compost cannot be isolated and cultured, their extracellular enzymes can be studied by analyzing their mRNA transcriptional information by using the metatranscriptomic method. If an mRNA transcriptional level of an enzyme in the thermophilic phase is significantly higher than the other phases, a plausible thermophilic enzyme could be discovered (Zhong et al. 2016). In this way, we discovered a glycoside hydrolase family 16(GH16) β-1,3-glucanase with highly catalytic activity at 75 °C. Its crystal structure shows two disulfide bonds, which plays an important role to stabilize the protein.

## Materials and methods

### Materials

The *E.coli* strain DH5α, Rosetta (DE3), and the high-fidelity ligase kit were purchased from TsingKe (Beijing, China). The plasmid extraction kit was purchased from MD Bio (Qingdao, China). The GelRed nucleic acid dye, the DNA Marker, the DNA Loading Buffer, and the protein Marker were purchased from Mingzhiyuan Health Management Co., Ltd. (Beijing, China). PEG3350 and the gem orange staining reagent were purchased from Sigma-Aldrich Trading Co., Ltd. (Shanghai, China). The curdlan substrate was purchased from Macleans Biochemical Technology Co., Ltd. (Shanghai, China). All other chemicals are of analytical grade.

### Bioinformatic analysis

The transcriptional levels of all mRNA sequences in the three phases of compost were analyzed using the metatranscriptomic method: The total RNA of compost was extracted using the RNA Isolation Kit (Qiagen) and sequenced for paired-end reads with Illumina HiSeq 3000/4000 workflow (Majorbio, Wuhan, China). The original sequence data were subject to quality control and assembled by the Trinity software (http://trinityrnaseq.github.io/, Version 2.13.1) (Grabherr et al. 2011). The Open Reading Frame (ORF) of each transcript was predicted by the TransGeneScan software (http://sourceforge.net/projects/transgenescan/, Version 1.2.1) (Ismail et al. 2014). The gene clustering was performed by the CD-HIT software (http://www.bioinformatics.org/cd-hit/) with 95% identity and 90% coverage. The longest gene of each cluster was selected as the standard sequence. The transcription level of each unigene was estimated by transforming the read density to fragments per kilo base of exon per million mapped reads (FPKM), and the function was annotated by BLAST against the NCBI non-redundant protein (NR) database.

When the transcriptional level of a particular enzyme in the thermophilic phase is significantly higher than the other two phases, we speculate it could be thermally stable and catalytically active. We analyzed their sequences using the PSI-Blast webserver (Oda et al. 2017). PSI-BLAST can analyze protein sequences using the Position Specific Scoring Matrix (PSSM) in an iteration manner, which could give better performance than the standard sequence alignment methods to find functional proteins (Jin et al. 2020). Among the enzymes, we found a putative β-1,3-glucanase. The phylogenetic tree of the enzyme was built using Mega7.0 and the diagram was depicted using the webserver ESPript3.0 (Kumar et al. 2016; Gouet et al. 1999). The theoretical molecular weight (MW) and isoelectric point (pI) were calculated using the webserver ProtPram (Artimo et al. 2012).

### Cloning, protein expression, and purification

The β-1,3-glucanase gene was codon optimized, synthesized, and cloned into the expression vector pCold II (Tsingke). The construct was transformed into the *E.coli Rosetta* (DE3) competent cells (Novagen) which are proper bacterial strains for the intact formation of disulfide bonds in cytoplasm (Zarkar et al. 2019). The protein was induced at 15 °C with 0.5 mM IPTG after 24 h cultivation in the minimal media, which could slow down protein folding and prevent formation of inclusion body during protein expression (Törnkvist et al. 1996). The harvested cells were lysed using a high-pressure cell press (Union Co., China) and clarified with high-speed centrifugation. The protein was purified using the nickel-affinity chromatography. The protein concentration was determined by measuring the absorbance at 280 nm (*ε*_280_ = 583,330 M^−1^ cm^−1^).

### Biochemical characterization

The enzyme activity was determined by the 3,5-dinitrosalicylicacid (DNS) method (Miller et al.^1959^) with minor revision: 2 mg/mL curdlan solution was heated for 5 min to obtain a pre-warmed suspension. 100 μl enzyme solution at the concentration of 0.004 mg/ml was added to the suspension and incubated for 10 min for reaction. The DNS solution was added and boiled for 10 min to terminate the reaction. The released reducing sugar after hydrolysis was measured by absorption at 540 nm.

We used different buffer solutions to measure the enzyme activity under different pH conditions (Additional file 1: Table S1). We incubated the enzyme solution at different temperatures for different time. After cooling for 4 min, the enzyme activity was analyzed to determine the thermal stability. The standard curve of D-glucose at the concentration of 0 ~ 0.8 μmol/mL was used to calculate the concentration of the reducing sugar. The V_max_ and K_m_ values were determined by fitting the Hill function of the reducing sugar using Origin 9.0 (Origin Lab, USA). The thermal stability of the β-1,3-glucanase was determined by measuring the residual enzyme activity at 70 and 75 °C for different time period at pH5.5. One activity unit (*U*) is defined when the enzyme releases 1 μmol reducing sugars per minute (Cheng et al. 2013).

### Crystallization, data collection, and structural refinement

β-1,3-glucanase was concentrated to about 20 mg/mL and was crystallized by the hanging drop method: 1 μL protein solution was added with 1 μL reservoir solution (21% PEG3350, 0.2 M magnesium chloride, 0.1 M Bis–tris, pH 5.5) and equilibrated with 0.5 mL reservoir solution at 18 °C. Crystals grew up in about 10 days and were dipped into the reservoir solution containing extra 25% glycerol as the cryo-protectant. After flash frozen, X-ray diffraction data were collected on the beamline BL18U1 of the Shanghai Synchrotron Radiation Facility (SSRF). Data reduction was performed using the HKL3000 program (Minor et al. 2006). The protein coordinates from 3DGT (Hong et al. 2008) with the sequence identity of 61% was used as the searching model for molecular replacement using the program MOLREP (Vagin and Teplyakov 1997). Refinements was carried out using REFMAC (Murshudov et al. 1997) implemented in the CCP4 program suite (Winn et al. 2011) with 5% of reflection reserved as the Free-R test set. Model building was manually carried out using Coot (Emsley et al. 2010). MolProbity was used to assess the structure quality (Chen et al. 2010). The reflection data and the crystal structure model were deposited in the Protein Data Bank (PDB ID: 7EO3).

### Molecular docking

The laminaritriose extracted from the crystal structure (PDB ID: 4BOW) (Labourel et al. 2014) is used as the ligand for molecular docking. A PDBQT file was prepared for the corresponding protein and ligand using AutodockTools 1.5.6. To prepare the PDBQT files for docking, essential hydrogen atoms and Kollman united atom charges are added using AutoDock Tools. The docking calculations are then performed with the AutoDock Vina program package (version 1.1.2) (Trott and Olson 2010). A grid box with the size of 35 × 35 × 35 Å points and the grid spacing of 0.375 Å has been generated using AutoGrid. The grid is centered at x, y, and z coordinates of 5.413, 12.734, and 32.776, respectively, which was reported as binding site of this enzyme (Labourel et al. 2014). The docking results were illustrated using PyMOL (DeLano Scientific) (Seeliger and de Groot 2010). The intermolecular interactions between protein and ligand were displayed using the LigPlot^+^ software (Laskowski and Swindells 2011).

### Differential scanning fluorometry

120 μL protein solution at the concentration of 0.5 mg mL^−1^ at pH5.5 was mixed with 0.8 μL SYPRO Orange Protein Gel Stain and was loaded to a 96-well PCR plate. Real-time PCR (Applied Biosystems 7300/7500, Thermo Fisher Scientific, USA) was performed to measure the temperature profile of protein unfolding (1 °C/min in the range of 25–98 °C). The fluorescence intensity was measured every 10 s. The Protein Thermal Shift™ software (version 1.4) was used to fit the original data and calculate the melting temperature (*T*_*m*_).

### Molecular dynamics (MD) simulations

MD simulations were carried out with GROMACS 5.1.4. The crystal structure of the catalytic domain (Actglu-CD) was used as the starting model with all the waters, ligands, and ions removed. The structures of C160G and C180I were obtained by point mutation using the crystal structure of Actglu-CD using the Modeller30 program (Webb and Sali 2016). The protein was dissolved in a cubic water tank filled with the TIP3P water molecules. Thirty-six sodium ions and twenty-five chloride ions were added to neutralize the system and simulate the physiological ionic strength of 0.15 M. PME was used to estimate static electricity under periodic boundary conditions. The AMBER99SB force field was used to simulate all elements in the cell, including proteins, salt ions, and water molecules. Before the MD simulation, the steepest descent method was used to minimize the energy, and then the basic Newton–Raphson method was used to eliminate the spatial collision and strain in the X-ray crystallographic structure. The LINCS algorithm was used to limit the bonds related to the hydrogen atoms, and the time step of the bond was 0.002 ps. A 100-ps NVT balance was performed, and the 100 ps NPT balance was performed at 370 K afterwards. Three independent runs of 300 ns starting with different velocity were performed.

## Results

### Bioinformatic analysis

**Fig. 1:**
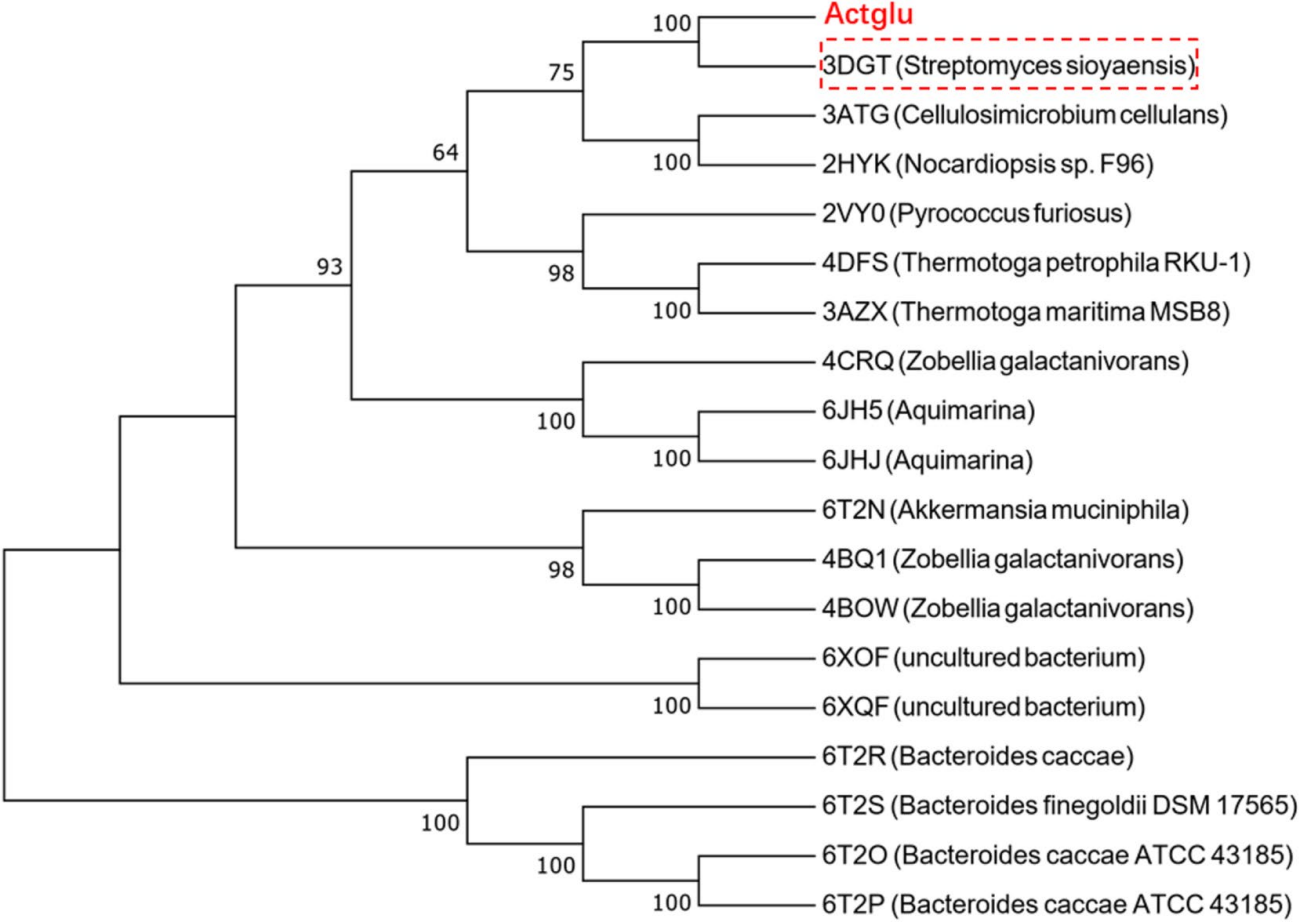
The phylogenetic tree of Actglu**.** The β-1,3-glucanase is most homologous with a GH16 family glucanase (PDB 3DGT) from *Streptomyces sioyaensis*, which belongs to *Streptomyces, Streptomycetaceae, Streptomycineae, Actinobacterales*

We analyzed the mRNA transcriptional level of all the proteins in compost using the metatranscriptomic method: We found that the transcriptional level of a putative β-1,3-glucanase in the thermophilic phase is significantly higher than that in the mesophilic and mature phases. The result indicates that this enzyme could be catalytically active at high temperatures. We named it as Actglu. Phylogenetic analysis indicates that this enzyme has the highest structural homology (61%) with a GH16 β-1,3-glucanase from *Streptomyces sioyaensis*, an actinobacterium (Fig. 1). Actglu’s amino acid sequence are deposited in GenBank with the accession number MZ366334.

**Fig. 2:**
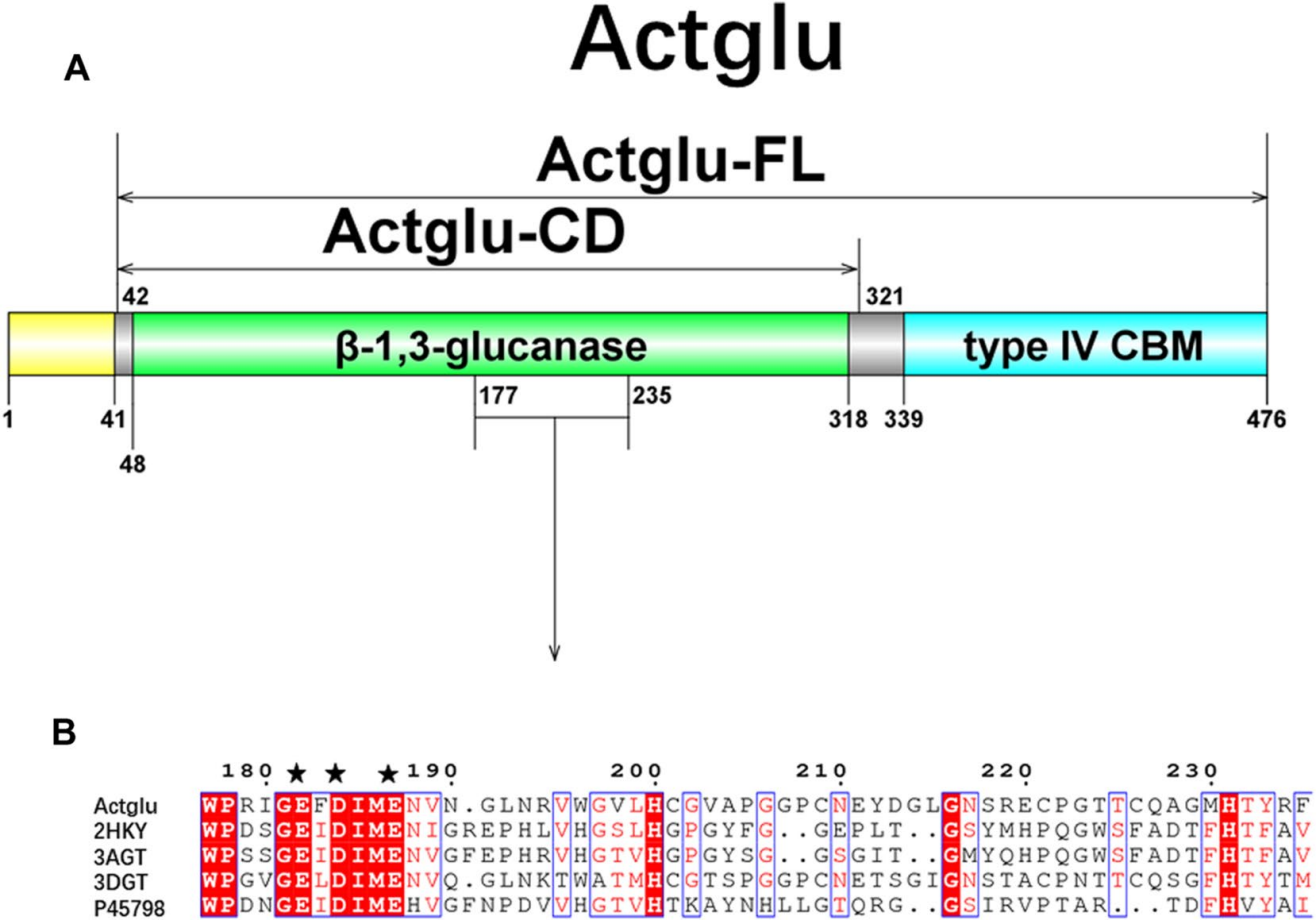
Schematic diagram of Actglu **A** and multiple sequence alignment of the active site (**B**). The yellow region is the signal peptide. The green region is the β-1,3-glucanase catalytic domain.The blue region is the type IV carbohydrate-binding domain (CBM). Multiple sequence alignment shows that Actglu has the conserved motif EXDXXE in the active site. The sequence marked with black five-pointed star is the conserved motif in the active site

The full length of enzyme (name as Actglu) has 476 amino acids. According to the sequence analysis of PSI-BLAST, it has a N-terminal signal peptide (residues 1 ~ 41), a β-1,3-glucanase catalytic domain (residues 48 ~ 318), and a type IV carbohydrate binding module (CBM, residues 339 ~ 473) (Fig. 2A). For further validation, we performed multiple-sequence alignment using this sequence with other GH16 family enzymes from different microorganisms, including *Streptomyces sioyaensis* (PDB ID: 3DGT), *Nocardiopsis sp. F96* (PDB ID: 2HYK), *Cellulosimicrobium cellulans* (PDB ID: 3ATG), *Rhodothermus marinus* (GenBank accession number: P45798). The alignment shows that the active site of this putative enzyme has the conserved motif EXDXXE, which is characteristic of GH16 family β-1,3-glucanases (Fig. 2B) (Ashida et al. 2002). We truncated the signal peptide and named the remaining region as Actglu-FL, which includes the catalytic domain and the CBM domain. The theoretical MW is 46.5 kDa and the pI is 4.7. We further truncated the CBM module to have the catalytic domain as Actglu-CD. Its theoretical MW is 30.7 kDa and its pI is 4.9, respectively.

### Expression, purification, and enzymatic characterization

The genes of Actglu-FL (including the catalytic domain and the CBM domain) and Actglu-CD (the catalytic domain) were cloned into the pCold II expression vector and transformed into the *E.coli* Rosetta(DE3) competent cells. Both proteins were induced by 0.5 mM IPTG at 15 °C for 24 h. After cell lysis and clarification, > 95% purity proteins were obtained by the Ni-affinity chromatography (Additional file 1: Figure S1).

**Fig. 3:**
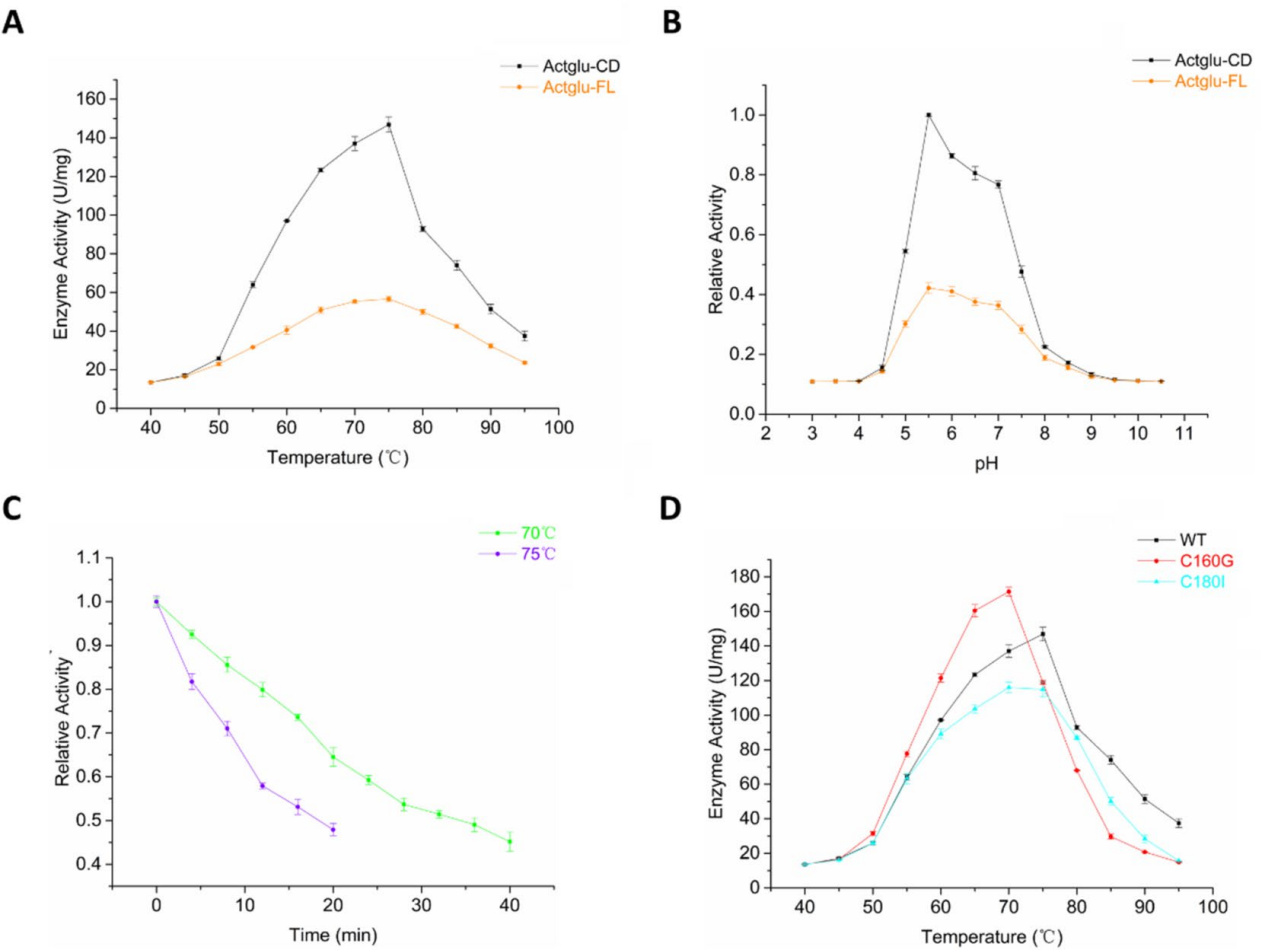
The activity and thermal stability of β-1,3-glucanase. **A** the temperature profile; **B** the pH profile; **C** the thermal stability of Actglu-CD at 70 and 75 °C, respectively. **D** activity comparison among Actglu-CD and its mutants (C160G and C180I)

The bell-shaped temperature profiles show that both Actglu-FL and Actglu-CD have the optimal reaction conditions at 75 °C and pH 5.5 (Fig. 3A, B). The enzyme activity of Actglu-CD is 146.9 U/mg in the optimal condition. Its half-life (*t*_1/2_) is about 35 min at 70 °C and about 19 min at 75 °C, respectively (Fig. 3C). In the optimal condition, its *V*_max_ is 677.2 μmol/min/mg and its *K*_*m*_ is 1.8 mg/mL, respectively (Table 1). In contrast, the activity of Actglu-FL is only one third of Actglu-CD (Fig. 3A). Thus, we only studied Actglu-CD in the following research, which we named as wild type (WT) β-1,3-glucanase henceforth in the text.

**Table 1:** Enzymatic kinetics of WT glucanase and its disulfide bond mutants

Enzyme	Temperature (℃)		*V*_max_ (μmol/min/mg)		*K*_*m*_ (mg/mL)	*k*_cat_ (s^−1^)	*k*_cat_/K_m_
WT		70		648.5	1.74	65.5	37.6
		75		677.2	1.80	68.4	38.0
C160G		70		717.0	1.81	72.4	40.0
		75		730.0	2.01	73.7	36.7
C180I		70		660.4	1.80	66.6	37.0
		75		687.1	1.96	69.4	35.4

### The crystal structure of the catalytic domain

**Fig. 4:**
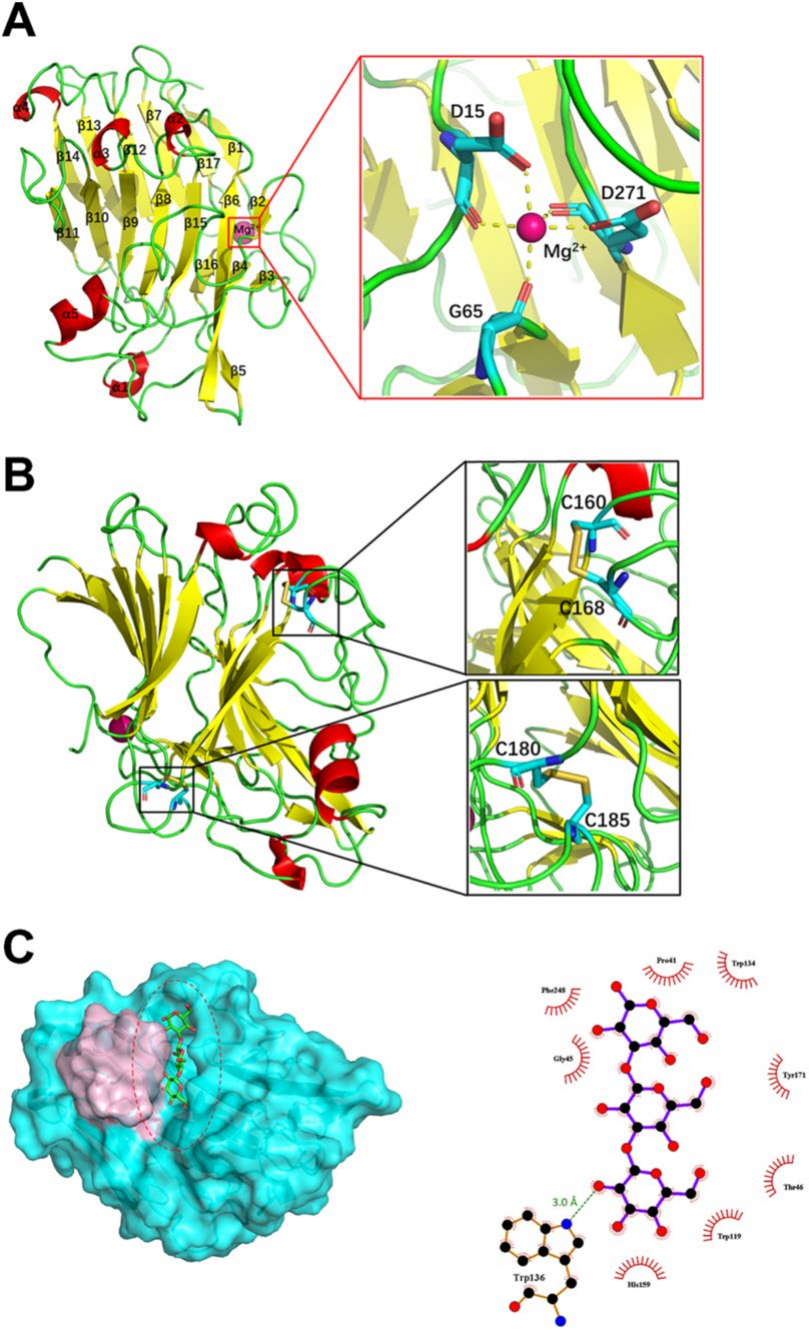
Structural analysis of the catalytic domain (Actglu-CD) of β-1,3-glucanase. **A** the overall structure. β-sheets, yellow; α-helix, red; Mg^2+^, bright pink; all loops, green. **B** two disulfide bonds in the structure (between C160 and C168, and between C180 and C185). The model in Panel B is rotated 90° around the Y-axis. **C** molecular docking shows the ligand binding cleft in Actglu-CD. The disulfide bonds are located in a region (light-pink, residues 156 –174) near the active site, which is illustrated by a red dashed oval

We crystallized the catalytic domain of β-1,3-glucanase by the hanging drop method. The space group is P2_1_2_1_2_1_. Crystal data were collected to 1.14 Å resolution. There is one molecule per asymmetric unit. We used the structure of an endo-1,3-β-glucanase from *Streptomyces sioyaensis* (PDB ID 3DGT) (Hong et al. 2008) with all waters, ligands, and ions removed as the initial template for molecular replacement using the program Phaser (McCoy et al. 2007) incorporated in the program suite Phenix (DiMaio et al. 2013). Actglu-CD and 3DGT have the sequence identity of 61%. After structure refinement using Phenix and manual model building using Coot (Emsley et al. 2010), the model has *R*_work_ of 0.132 and *R*_free_ of 0.158, respectively (Table 2). The overall structure shows a sandwich-like β-jelly-roll fold, which is typical for GH16 enzymes (Fig. 4A) (Dong et al. 2015). The overall structure includes seventeen β-strands, one α-helix, and four short 3_10_-helices. β-1,3-glucanase uses the two-step retaining mechanism for hydrolysis (Vuong and Wilson 2010). The catalytic site contains Glu141 that acts as the nucleophile and Glu146 that plays the role as the acid/base (Ashida et al. 2002). Near the N-terminus of the protein, a putative magnesium ion binds to the oxygen atoms of the carbonyl main chain and the carboxylate side chain of Asp15, Gly65, and Asp271 to form an octahedral geometry (Fig. 4A). There are two disulfide bonds formed by Cys160 and Cys168, and by Cys180 and Cys185 (Fig. 4B), which possibly stabilize the protein fold at high temperatures (Dehnavi et al. 2017).

**Table 2:** Diffraction data collection and refinement statistics

Variable	X-ray 100 K
Data collection	
Space group	P 2_1_2_1_2_1_
Unit cell dimensions	
a, b, c (Å)	60.4, 61.0, 70.7
α, β, γ (°)	α = β = γ = 90°
Resolution (Å)	50.00–1.14 (1.16–1.14)
Unique reflections	95,197 (4703)
Multiplicity	11.5 (8.3)
Completeness (%)	100.00 (100.00)
Wavelength (Å)	0.98
R_sym_^a^	0.150 (0.942)^b^
< I > /σ < I >	16.6 (2.0)
Refinement	
Resolution (Å)	16.93–1.14 (1.16–1.14)
R_work_^c^/R_free_^d^	0.132/0.158
No. of atoms	
Protein	2221
Tris	8
Mg^2+^	1
Water molecules	297
B factors	
Protein	12.29
Solvent	26.84
Deviation from ideality	
Bond length (Å)	0.0136
Bond angle (°)	1.842
Ramachandran plot statistics (%)	
Preferred regions	97.86
Allowed regions	2.14
Outliers	0
PDB ID code	7EO3

^a^R_sym_ = ∑ (|*Ii* – < *I* >|)/∑(*I*), where *Ii* is the measured intensity and < *I* > is the mean intensity of all measured observations equivalent to reflection *Ii*

^b^Values in parentheses are statistics from the highest-resolution shell

^c^R_work_ = ∑||*F*_obs_| – |*F*_calc_||/∑|*F*_obs_|, where |*F*_obs_| is the observed diffraction amplitude and |*F*_calc_| is the corresponding calculated structure factor amplitude

^d^R_free_ is defined as R_work_, which involves 5% of the measured reflections not used in refinement and set aside for cross-validation

The docking study shows that the binding free energy of laminaritriose with Actglu-CD is -420 kcal/mol, which indicates that the ligand binds tightly in the active site cleft (Fig. 4C). The ligand has hydrophobic interactions with residues in the cleft, such as Trp134, His159, Tyr171 and Phe248. There is a hydrogen bond between laminaritriose and Trp136. The above interactions could contribute to its tight binging affinity (Fig. 4C) (Fibriansah et al. 2007; Ashida et al. 2002).

### Functional analysis of the two disulfide bonds

**Fig. 5:**
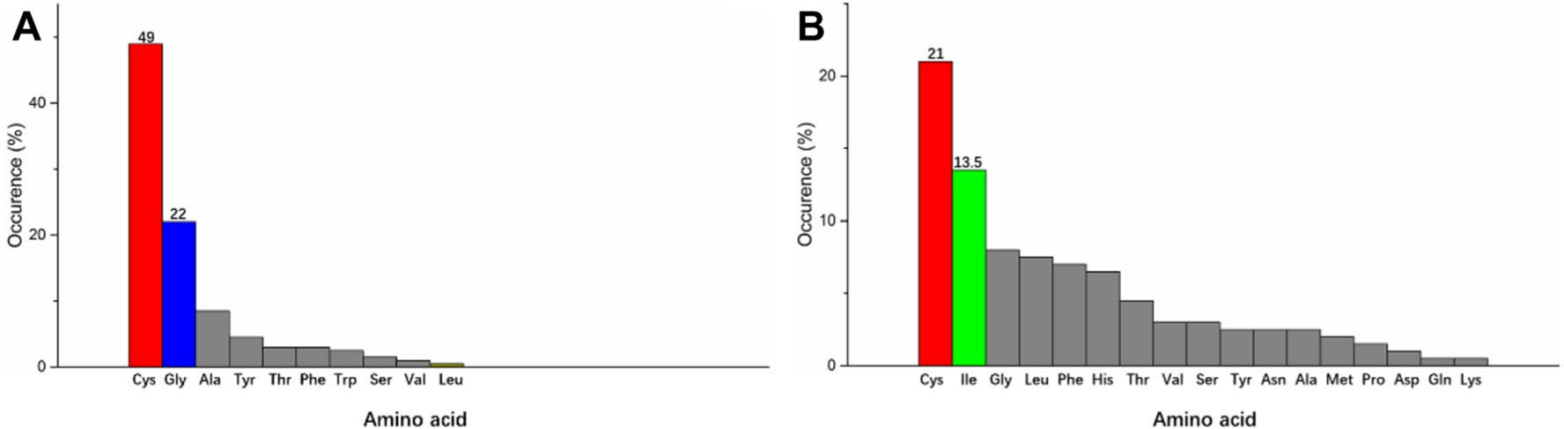
Occurrence of different amino acids at the residue positions of 160 and 180, respectively. Glycine is the second most occurred residue at the position 160 (**A**), and isoleucine is the second most occurred residue at the position 180 (**B**)

To validate the consolidating effect afforded by the disulfide bonds, we performed mutagenesis to break them. We used the HotSpot Wizard webserver to analyze the occurred frequencies at the residue positions 160 and 180. The results show that glycine is the second most prevalent residue at position 160, and isoleucine is the second most prevalent residue at position 180 (Fig. 5). Thus, we designed two single mutants, C160G and C180I, respectively. C160G has the bell-shaped temperature profile with the maximum activity at 70 °C, which is 5 °C lower than that of wild type (WT). In the temperature range of 50 ~ 70 °C, its activity is significantly higher than that of WT (Fig. 3D). Specifically, the activity of C160G at its optimal temperature is 171.4 U/mg, which is 17% higher than that of WT. The catalytic efficiency (*k*_cat_/*K*_*m*_) in its optimal condition is also higher than that of WT (Table 1). C180I also has the bell-shaped temperature profile with the peak activity at 70 °C. However, the activity of C180I is lower than that of the WT (Fig. 3D). We compared the half-lives (*t*_1/2_) of WT and the two mutants at both 70 and 75 °C. The results show that WT is most stable, C180I is the second, and C160G has the least thermal stability (Table 3).

**Table 3:** Half-life (*t*_1/2_) of WT and its two mutants

Enzyme	Temperature (℃)	*t*_1/2_ (min)
WT	70	35
	75	18
C160G	70	16
	75	7
C180I	70	28
	75	12

**Fig. 6:**
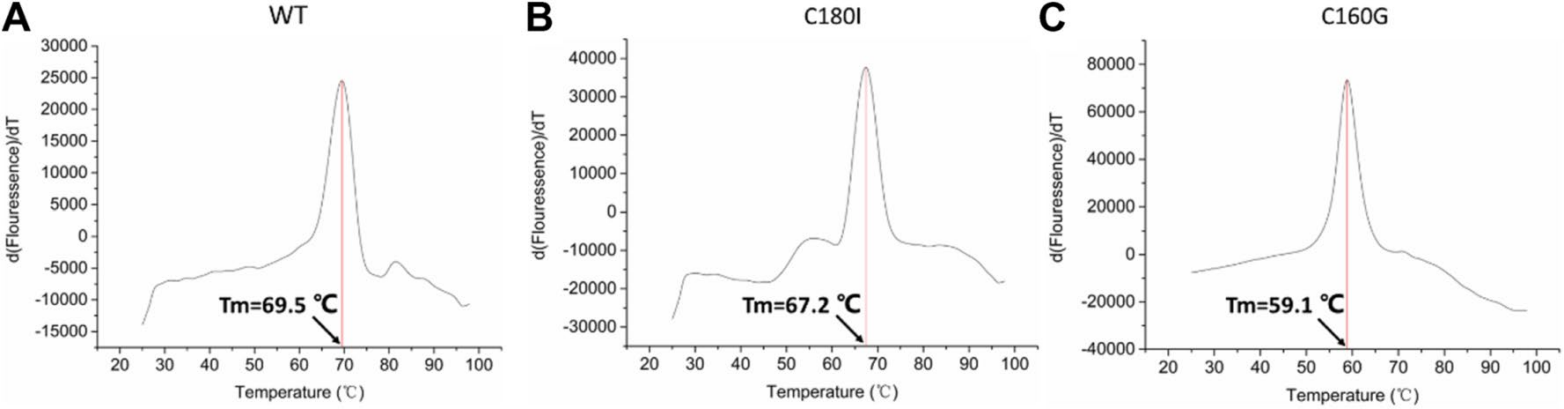
Tm values of WT, C160G, and C180I. The *T*_*m*_ values are 69.5 °C for WT (**A**), 67.2 °C for C180I (**B**), and 59.1 °C for C160G (**C**), individually

We used the differential scanning fluorometry to study the melting temperature (*T*_*m*_) of WT and its mutants. The *T*_*m*_ value is 69.5 °C for WT, 67.2 °C for C180I, and 59.1 °C for C160G, individually (Fig. 6). The results confirm that WT is the most stable, C180I is the second, and C160G is the least.

### Molecular dynamics simulations.

**Fig. 7:**
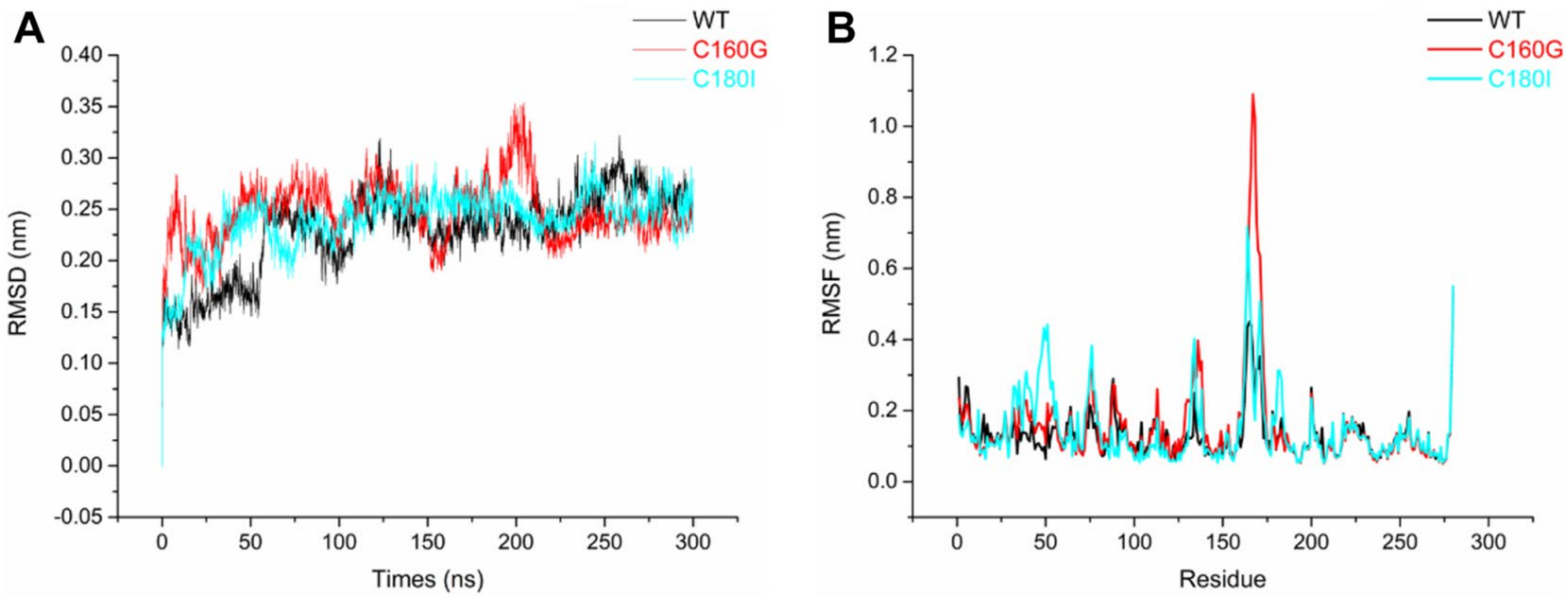
Molecular dynamics simulations reveal the dynamic differences among WT and its mutants. **A** After 300 ns, all the proteins are in equilibrium with similar RMSD. **B** RMSF comparison shows that the loop region (residues 156–174) in C160G is most dynamics, and the region in WT is most rigid

Our MD simulations show that after 300 ns, WT and the mutants have been equilibrated without significant conformational changes (Fig. 7A). However, RMSF comparison shows that the loop region (residues 156–174) in C160G is most dynamic, whereas this region in WT is most rigid (Fig. 7B). This loop region is close to the substrate binding cleft (Fig. 4C). Thus, abolishing the disulfide bonds increases flexibility near the active site, which could influence substrate binding and product release for catalysis.

## Discussion and conclusion

Microorganisms in the thermophilic phase of compost can produce a large amount of thermostable enzymes for rapid degradation of organic waste (Wang et al. 2021). Here, we discovered a thermophilic β-1,3-glucanase from compost using the metatranscriptomic method. The full length enzyme includes a signal peptide region, a catalytic domain (CD), and a carbohydrate-binding module (CBM) (Fig. 2A). Phylogenetic analysis indicates that the exolytic enzyme is homologous with a β-1,3-glucanase from the genus *Streptomyces sioyaensis* (sequence identity 61%). Multiple sequence alignment shows that it has the conserved catalytic motif EXDXXE of the GH16 family β-1,3-glucanase. Previous studies showed that these glutamates are very conservative and their corresponding mutants have almost lost all enzyme activity (Hahn et al. 1995; Labourel et al. 2014). The activity of the Actglu-FL (the catalytic domain appended with the carbohydrate-binding module) is only one third of the catalytic domain (Actglu-CD). This could be that curdlan used in our experiments as the substrate is not crystalline. CBM can bind to the surface of substrates and break up their crystalline structure to promote hydrolysis (Shoseyov et al. 2006). Thus, CBM in the full length enzyme does not play a role in our experiments and we only studied the structure and activity of the catalytic domain (named as Actglu-CD).

The crystal structure of Actglu-CD shows that the protein has a sandwich-like β-jelly-roll fold, which is typical of the GH16 family β-1,3-glucanases. There are two disulfide bonds in the structure (between C160 and C168 and between C180 and C185), which stabilize the protein fold. The catalytic activity of this enzyme is 146.9 U/mg under the optimal condition (75 °C, pH 5.5) with curdlan as the substrate. It has the sequence identity of 61% with a well characterized β-1,3-glucanase (PDB ID: 3DGT), which has the activity of 19.0 U/mg. (Hong et al. 2002). Compared with the crystal structure of Actglu-CD, 3DGT has one more β-strand and one more α-helix (Hong et al. 2008). Previous studies have shown that improvement of thermal stability can be achieved by stabilizing secondary structures such as α-helix and β-sheet (Jaenicke et al. 1996). As a result, 3DGT has longer half-life at 75 °C (Hong et al. 2008).

Many studies showed that disulfide bonds are essential to maintain the structural stability of proteins (Dehnavi et al. 2017; Yennamalli et al. 2011). To validate their role in Actglu-CD, we performed mutagenesis to abolish them. We evaluated the occurring frequencies at the residue positions 160 and 180 using HotSpot Wizard 3.0 and chose the second most occurred residues (Fig. 5). Accordingly, we designed C160G and C180I. As expected, abolishing the disulfide bonds decreases the structural stability. Compared with wild type (WT), the T_m_ values of C160G and C180I have decreased by 10.4 °C and 2.3 °C, respectively. Their optimal reaction temperatures have decreased by 5 °C. Their t_1/2_ is also significantly decreased at 70 °C and 75 °C. Interestingly, C160G has higher activity in the temperature range of 50 ~ 70 °C (Fig. 3D). Our molecular dynamics simulations show that the loop region (residues 156 ~ 174) in C160G is significantly more flexible than WT, which is near the active site (Figs. 4C and 7B). The increased dynamics may have two effects. First, it may lead to lower substrate binding affinity to have higher K_m_ values. Second, the increased conformational flexibility may decrease the product binding affinity to accelerate its release, which is usually the rate-limiting step in enzyme catalysis (Saavedra et al. 2018). Thus, k_cat_ of C160G is significantly increased and the overall catalytic efficiency (*k*_cat_/*K*_*m*_) is increased at 70 °C (Table 1). H.G. Saavedra et al. shows that mutating a ‘heavy’ surface residue to a ‘lighter’ residue (Glycine) has increased dynamics in the local region, which could propagate to the active site to lower the activation barrier and promote catalysis (Saavedra et al. 2018; Jeng et al. 2011). Our results are consistent with their findings. In contrast, there are hydrophobic amino acids near C180, such as proline and alanine. When C180 is mutated to *I*, it may have hydrophobic interactions with its surrounding residues. These interactions would lead to only slightly increased dynamics compared with WT (Fig. 7B), and gives rise to similar *k*_cat_ compared with WT. However, C180I has higher *K*_*m*_. Consequently, its overall catalytic efficiency (*k*_cat_/*K*_*m*_) is lower than that of WT (Table 1 and Fig. 3D).

Mutating residues in the active site to increase enzyme activity is difficult to be successful, because such mutations could jeopardize the conserved conformation of the catalytic residues (Fields and Somero 1998). Mutating residues distal to the active site, particularly in the loop region on the surface, would have the least conformational changes. Exolytic enzymes from different microorganisms use this strategy to adapt to different temperature environments (Fields et al. 2015). Here, we have designed mutations in β-1,3-glucanase based on natural evolution, which led to decrease of its thermal stability, but increase of its catalytic activity. This strategy could be useful to adjust the enzyme activity according to different applications in industry.

### Supplementary Information

**Additional file 1. Figure S1.** SDS-PAGE analysis of the full length (Actglu-FL), the catalytic domain (Actglu-CD), and the mutants. **Table S1.** The buffer solutions used under different pH conditions.

## Data Availability

All data generated or analyzed during this study are included in this article.
