# Supplementary material for: Identification and structural analysis of a thermophilic β-1,3-glucanase from compost
Source: Bioresour Bioprocess. 2021 Oct 17;8(1):102. doi: 10.1186/s40643-021-00449-4 (PMC10992293; doi:10.1186/s40643-021-00449-4)
Supplement: Supplementary file 1 — Additional file 1. Figure S1. SDS-PAGE analysis of the full length (Actglu-FL), the catalytic domain (Actglu-CD), and the mutants. Table S1. The buffer solutions used under different pH conditions. [file 40643_2021_449_MOESM1_ESM.docx]

**Identification and structural analysis of a thermophilic β-1,3-glucanase from compost**

Jianwei Feng^1^, Shenyuan Xu^3^, Ruirui Feng^1^, Andrey Kovalevsky^4^, Xia Zhang^5^, Dongyang Liu^6^, Qun Wan^1,2，^

^1^ College of Science, Nanjing Agricultural University, Nanjing 210095, People’s Republic of China

^2^ Key laboratory of Plant Immunity, Nanjing Agricultural University, Nanjing 210095, People’s Republic of China

^3^ Key Laboratory of Bioorganic Synthesis of Zhejiang Province, College of Biotechnology and Bioengineering, Zhejiang University of Technology, Hangzhou, P.R. China

^4^ Neutron Scattering Division, Oak Ridge National Laboratory, Oak Ridge, Tennessee 37831, United States of America

^5^ Department of Molecular Biology, Qingdao Vland Biotech Group Inc., Qingdao, Shandong 266000, People’s Republic of China

^6^ College of Resources and Environmental Sciences, Nanjing Agricultural University, Nanjing 210095, People’s Republic of China

*Correspondence: qunwan@njau.edu.cn

**Additional file 1**

① ② ③ ④ ⑤


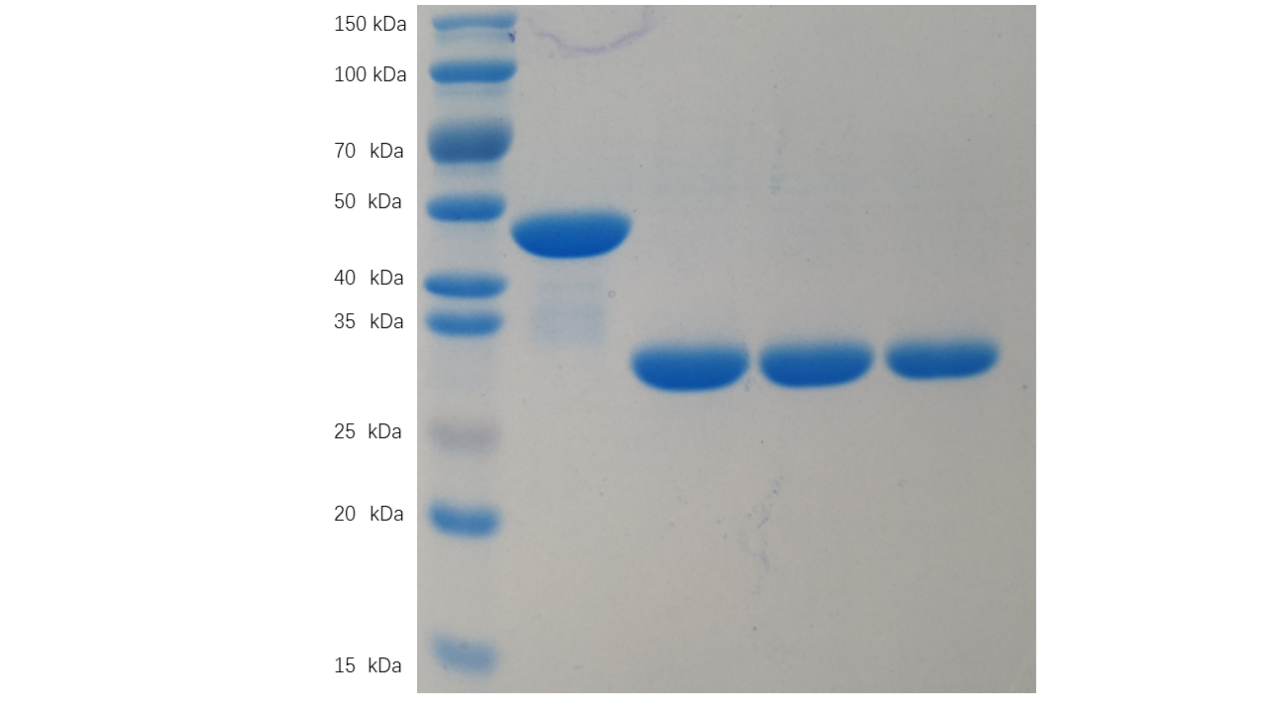


**Fig. S1** SDS-PAGE analysis of the full length (Actglu-FL), the catalytic domain (Actglu-CD), and the mutants. ①protein marker， ②Actglu-FL，③Actglu-CD, ④the C160G mutant, ⑤ the C180I mutant.

**Table S1. Buffer solution used under different pH conditions**

| **pH** | **Buffer solution** | **Concentration** |
| --- | --- | --- |
| 3.0-3.5 | Citric acid-sodium citrate | 0.1 M |
| 4.0-5.5 | Acetic acid-sodium acetate | 0.1 M |
| 6.0-6.5 | MES | 50 mM |
| 7.0-9.0 | Tris | 50 mM |
| 9.5-10.5 | Glycine-sodium hydroxide | 50 mM |
